# High-expression of ROCK1 modulates the apoptosis of lens epithelial cells in age-related cataracts by targeting p53 gene

**DOI:** 10.1186/s10020-020-00251-6

**Published:** 2020-12-09

**Authors:** Shanshan Hu, Dongmei Su, Lei Sun, Zhongying Wang, Lina Guan, Shanhe Liu, Baowen Zhao, Yong Liu, Cuige Shi, Jianbo Yu, Xu Ma

**Affiliations:** 1 Hongqi Hospital of Mudanjiang Medical College, 5 Tongxiang Road, Mudanjiang, 157011 Heilongjiang China; 2 Department of Genetics, National Research Institute for Family Planning, Health Department, Beijing, 100081 China; 3 Medical Research Center of Mudanjiang Medical College, Mudanjiang, 157011 Heilongjiang China; 4 Graduate School, Peking Union Medical College, Beijing, 100081 China; 5 Department of Immunology, Basic Medical College of Harbin Medical University, Harbin, 150081 Heilongjiang China

**Keywords:** Age-related cataract, Lens epithelial cells, Apoptosis, ROCK1, P53

## Abstract

**Background:**

Age-related cataract (ARC) is a serious visual impairment disease, and its pathogenesis is unclear. This article aims to investigate the role of ROCK1 in the apoptosis of lens epithelial cells (LECs) in age-related cataracts.

**Methods:**

We collect anterior capsule samples from normal people, patients with age-related cataracts, young mice and naturally aging cataract mice. The oxidative stress-induced apoptosis model was constructed by cultivating HLE-B3 cells with H_2_O_2_. MTT, Hoechst 33342, and TUNEL assay were performed to explore proliferation and apoptosis. HE assay was used to observe cell morphology. The gene and protein expression were assessed by quantitative real-time PCR, western blot, immunofluorescence, and immunohistochemical staining.

**Result:**

The results from the clinic and mice experiments showed that the numbers of lens epithelial cells from cataract individuals were less than the control individuals. In vitro, the apoptotic cells were increased in lens epithelial cells under H_2_O_2_ treatment. The ROCK1 protein level increased in the lens epithelial cells from age-related cataract patients and the old mice, respectively. Meanwhile, the up-regulation of the ROCK1 gene was associated with H_2_O_2_-induced HLE-B3 cells apoptosis. MTT and apoptosis assay showed ROCK1 was necessary in mediating H_2_O_2_-induced lens epithelial cells apoptosis through ROCK1 over-expression and knockdown experiment, respectively. Further investigation showed that p53 protein levels had been increased during ROCK1-mediated apoptosis in response to H_2_O_2_. Besides, ROCK1 phosphorylated p53 at ser15 to up-regulate its protein level.

**Conclusions:**

This study established the novel association of ROCK1/p53 signaling with lens epithelial cells apoptosis and age-related cataract genesis.

## Background

Cataracts cause blindness through the opacification of the ocular lens, and they are the single most common cause of blindness worldwide. At least 42% of cases of blindness are caused by cataracts. According to the World Health Organization, more than 40 million people worldwide will become blind due to cataracts by 2020 (Gao et al. 2015). With the aging population increasing, the incidence of age-related cataracts (ARC) is likely to further increase. Surgery is currently the only effective means of treating cataracts, but it brings about a heavy financial burden for patients in developing countries (Ravindran et al. 2019; Wang et al. 2020). Thus, in-depth studies and illumination of ARC' development and the specific molecular mechanisms in its developing process are to find the causes of the disease and develop effective anti-cataract medications, and to prevent, delay, or even reverse the lens opacity, which can protect the patients' visual function to the maximum and improve their quality of life. It has great theoretical and social significance in reducing patients' and social burden.

Currently, the exact cause of ARC has not been fully established, and oxidative stress damages lens protein and cause lens epithelial cell apoptosis, which is considered to be the common molecular basis for the development of cataract (Su et al. 2017a). Apoptosis, also known as programmed cell death, is a normal physiological phenomenon in certain stages of animal development (Li et al. 1995). Previous studies have been reported that H_2_O_2_ is the main reactive oxygen species (ROS) that exists in the lens of the eye. Long-term exposure to H_2_O_2_ can lead to lens morphology and cataract (Wang et al. 2018). Specific cellular signaling pathways during oxidative stress-induced apoptosis of lens epithelial cells remain unclear.

Rho-associated kinase, also known as Rho-associated coiled-coil forming protein serine/threonine kinase (ROCK), is a member of protein kinase A, G, and C (PKA/PKG/PKC) families. ROCK has two highly homologous isoforms, ROCK1, and ROCK2, which are 65% identical in amino acid sequence and up to 92% identical in the kinase domain (Nakagawa et al. 1996). However, ROCK1 and ROCK2 have functionally different roles in regulating cell adhesion and cell death under stress conditions (Surma et al. 2014). Michaël et al. found that ROCK1 is sensitive to caspase-3 mediated apoptosis, and ROCK2 does not have this function (Sebbagh et al. 2001). Some publications suggested that RhoA/ROCK plays an important molecular "switch" in eye disease. According to the report, ROCK participates in the migration of rabbit corneal epithelial cells, increases the aqueous humor outflow pathway through inhibitors (Nakamura et al. 2001; Rao et al. 2001), and induces cytoskeletal recombination of lens epithelial cells and epithelial–mesenchymal transition (Korol et al. 2016; Imaizumi et al. 2019). However, little is known about the expression of ROCK1 under oxidative stress and its role in lens epithelial cells apoptosis and the development of ARC.

Hence, this study aims to investigate the expression pattern of ROCK1 and related molecular mechanisms in H_2_O_2_-mediated apoptosis in lens epithelial cells (LECs) and pathogenesis of age-related cataract by cytologic study, mice models and clinic samples (the central area of the anterior lens capsule) studies. Based on the experimental data generated from this study, we established the association ROCK1 up-regulation with LECs apoptosis and ARC. Further studies showed that ROCK1 phosphorylated p53 at ser15 to up-regulate its protein level to mediate LECs apoptosis. This study demonstrates a novel role of ROCK1/p53 signaling in modulating H_2_O_2_-induced LECs apoptosis and in vitro and in vivo.

## Materials and methods

### Study participants and preparation of the central area of the anterior lens capsule

We selected cortical cataract (the most common clinical phenotype of ARC) patients as the study subjects. A total of 30 eyes of patients (male: 13 cases of 13 eyes, female: 17 cases of 17 eyes) with age-related cortical cataract treated by operation in our hospital from January 2016 to December 2017 were collected under the following standards: (1) the age ranged from 55 to 65; (2) non-congenital cataract, non-metabolic cataract, and non-secondary cataract; (3) without hypertension, diabetes mellitus, fundus lesions, uveitis, and glaucoma; (4) no eye trauma and the history of intraocular surgery. In the study, the normal subjects (the total of 30 eyes, male: 18 cases of 18 eyes, female: 12 cases of 12 eyes) with transparent lens were enrolled from corneal transplant donors and ocular trauma patients with lens detachment, which the age range was from 46 to 55. We declared that the study followed the tenets of the Declaration of Helsinki, and was approved by the Ethics Committee of Mudanjiang Medical University. All subjects in this study knew and understood the content and risk of the research and signed the informed consent.

The central area of the anterior lens capsule (including normal people and ARC patients) were obtained by a single ophthalmologist during surgery using the intact continuous curvilinear capsulorhexis method. After the tissue samples were separated, it was immediately placed in 4% paraformaldehyde or liquid nitrogen. Tissue samples placed 4% paraformaldehyde at room temperature were used for HE analysis and immunohistochemical staining, while those stored in liquid nitrogen were used for protein and RNA extraction.

### Establishment of animal model and isolation of lenses

The animal model is based on our previous research (Su et al. 2017a) and has been modified. 30 Young (3 months) and 30 old (> 24 months) male BALB/c mice were housed with free access to food and water at a mean ± SD constant temperature of 22 ± 2 ℃, the humidity of 55 ± 5%, and a 12 h light/12 h dark cycle, and their eyes were observed daily. Partial opacity of lens was observed over 24 months, and the eyes photos were taken. This study was performed in strict accordance with the recommendations in the Guide for the Care and Use of Laboratory Animals of the National Institutes of Health. The experimental protocol was approved by the National Institutes of Health Guide for Care and Use of Laboratory Animals.

The mice were euthanized by cervical dislocation. The entire eye of each mouse was removed, its cuticles were placed on sterile gauze face down, and fixed with tweezers. An incision is then made on the of the optic nerve that enters the eye, and the sclera is pulled back to expose the lens. Before staining, use a pair of tweezers to gently lift the ciliary body fragments attached to the equator plane of the lens.

### Hematoxylin and eosin (H&E) and terminal deoxynucleotidyl transferase dUTP nick end labeling (TUNEL) assay

Samples were fixed with 4% paraformaldehyde and embedded in paraffin, and the thickness of the sections was 4 μm. The prepared paraffin sections were dewaxed and hydrated with xylene and graded alcohols. The sections were washed with phosphate-buffered saline (PBS, South Logan, UT, USA), and then stained with hematoxylin and eosin, and images were captured using Nikon Eclipse microscope.

In the TUNEL assay, a kit (Roche, Basel, Switzerland) was used for in situ cell death detection according to the manufacturer's instructions. Briefly, sections were incubated with TUNEL labeling solution at 37 ℃ for 1 h and stained with Hoechst 33342 (Sigma-Aldrich, St. Louis, MO, USA). Sections were analyzed with a Nikon Eclipse fluorescence microscope.

### Immunohistochemical (IHC) staining

The specific method of immunohistochemical (IHC) staining has been described in our previous article (Su et al. 2017b). After paraffin sections were incubated at 60° for 2 h, they were dewaxed and hydrated with xylene and graded alcohol. After washing three times with PBS, it was boiled in 0.1 M citric acid (pH 6.1) for 30 min and cooled to room temperature. PBS has washed again and soaked with 0.3% H_2_O_2_ to inhibit endogenous peroxidase activity. Sections were incubated with normal fetal bovine serum (FBS, GIBCO, Grand Island, NY, USA) for 30 min and then treated with anti-ROCK1 (1:1000, Abcam), anti-p53 (1:500, Cell Signaling Technology), and anti-phospho-p53 (1:20, Abcam) antibodies overnight. The next day, the corresponding secondary antibody conjugated to horseradish peroxidase were incubated for 1 h. Sections were incubated in a peroxidase substrate solution (diaminobenzidine hydrochloride, DAB, Cell Signaling Technology, Danvers, MA, USA). The control group and the experimental group were simultaneously dripped with an equal amount of DAB solution. After coloring, the two were placed in PBS at the same time to stop the color development, to ensure the same staining time, and avoid the differential expression of results due to the time interval. After that, xylene and fractionated alcohol were dehydrated again, and then the sample was covered with paramount. Immunostaining images were captured using a Nikon Eclipse microscope.

### Western blotting

Frozen lens tissues from humans and mice were extracted in RIPA lysed buffer with protease inhibitor cocktail (Pierce, USA). Total protein (40 µg) was applied to a 10% SDS-polyacrylamide electrophoresis gel. We use molecular mass marker protein standard (Biotides, China) as a guide, and cut the gel horizontally to approximate the size of the protein to contain the protein of interest. To save protein, we cut different blots on the same group of protein gels to detect proteins of different molecular weight. The polyvinylidene fluoride (PVDF) membrane (Millipore, Billerica, MA, USA) was incubated with the primary antibody. Although Western blotting uses the same antibodies as IHC, the use ratio has changed, as follows: anti-ROCK1 (1:2000), anti-p53 (1:500), anti-phospho-p53 (1:100) and anti-β-actin (1:1500, Sigma-Aldrich, St. Louis, MO, USA). Observation of immune response bands by chemiluminescence substrate method with a super signal western pico kit (Pierce Co, USA). Three independent experiments were performed. Protein quantitative was analyzed using Image J software.

### Cell culture and plasmids

Human lens epithelial B3 (HLE-B3) cells were donated by Professor Qi (Harbin Medical University) and cultured in Dulbecco's modified Eagle's medium (DMEM, GIBCO, Grand Island, NY, USA) with 20% FBS, and penicillin–streptomycin (1:100, Sigma-Aldrich, St. Louis, MO, USA) in a humidified atmosphere containing with 5% CO_2_ at 37 ℃. ROCK1 over-expression plasmid (pcDNA-ROCK1) and pcDNA negative control (pcDNA-NC) were purchased from the Polepolar Research Company (China). The CDS region of p53 is constructed to the GFP vector. The small interfering RNA (siRNA) targeting sequences of ROCK1 (si-ROCK1) was 5′-GAAGAAACATTCCCTATTC-3′ (Liebig et al. 2009), and the sequences of si-p53 was 5′-GACTCCAGTGGTAATCTAC-3′ (Ding et al. 2007). The negative control siRNA (si-NC) sequence was 5′-CGTCAACATGGCTTTCACC-3′. The si-ROCK1 and si-p53 plasmids were constructed as we did in the previous articles (Su et al. 2017b).

### H_2_O_2_ treatment and transfection

Human lens epithelial cells B3 cells at 80% confluency were treated with H_2_O_2_ at the indicated concentrations (200 µM). Transient transfection B3 cells were performed using the Lipofectamine 3000 (Invitrogen). DNA plasmids were transfected into B3 cells by using lipofectamine 3000 under manufacture procedures. Cells were starved when transfected with a plasmid (1.25 µg/ml), H_2_O_2_ (200 µM) was added after 1 h and 20% FBS was added 4 h later.

### MTT assay

As our previous study [4], the 3- (4,5-dimethylthiazole-2-yl) -2,5-diphenyltetrazole ammonium bromide (MTT) assay was used to determine the cell survival rate. After treatments or transfection, cells were incubated with 20 µl MTT (5 mg/ml) solution for 4 h. Then, the medium was aspirated and cells were dissolved with 150 µl of dimethyl sulfoxide. The absorbance was examined with a microplate reader at 490 nm.

### Apoptosis assay

Hoechst 33342 (Sigma-Aldrich, St. Louis, MO, USA) was used to observe the morphological changes of the nucleus and evaluate the apoptosis. Briefly, cells were cultured in a 6-well plate for a certain period time and then stained with Hoechst 33342. Images were selected using a fluorescence microscope, 5 fields were randomly selected to count the number of apoptotic nuclei.

### Immunocytochemistry

The B3 cells were cultured in 24-well plates and treated with 200 µM H_2_O_2_ in media for 24 h. These cells were fixed with 4% paraformaldehyde for 10 min, permeabilized in 0.3% Triton X-100 for 10 min, blocked with 3% bovine serum albumin (BSA) for 30 min at 37 ℃. The primary antibodies of ROCK1 (1:200, Abcam) and p53 (1:200, Cell Signaling Technology) were first incubated at 37 ℃ for 1 h and then at 4 ℃ overnight. Alex Fluor 594 goat anti-rabbit.

IgG (Invitrogen, A11037) and Alex Fluor 488 goat anti-mouse IgG (Invitrogen, A11029) were as the secondary antibody incubated for 1 h at 37 ℃. Images were selected using a fluorescence microscope.

### RNA extraction, reverse transcription, and quantitative RT-PCR

Total cellular RNA was extracted from B3 cells using the Promega Total RNA Isolation System according to the manufacturer’s instructions. Reverse transcription-polymerase chain reaction (RT-PCR) was performed using the Access RT-PCR System (Promega). The ROCK1 gene primer pairs for quantitative PCR assay were as follows: sense, 5′-TGTGACTGGTGGTCGGTT-3′ and antisense, 5-′GGTTTTTTGCTTCTTTT′G-3. Primer pairs for β-actin were as follows: sense, 5′-TCGTGCGTGACATTAAGGAG-3′, and antisense, 5′-ATGCCAGGGTACATGGTGGT′-3.

### Statistical analysis

Student t-test and ANOVA analyses were used to calculate the statistical significance of the experimental data. Significance level were set as *p < 0.05; ^#^p < 0.05; **p < 0.01; ^##^p < 0.01. Error bars denote SD.

## Results

### Lens epithelial cells apoptosis was associated with the age-related cataract base on human samples

**Fig. 1:**
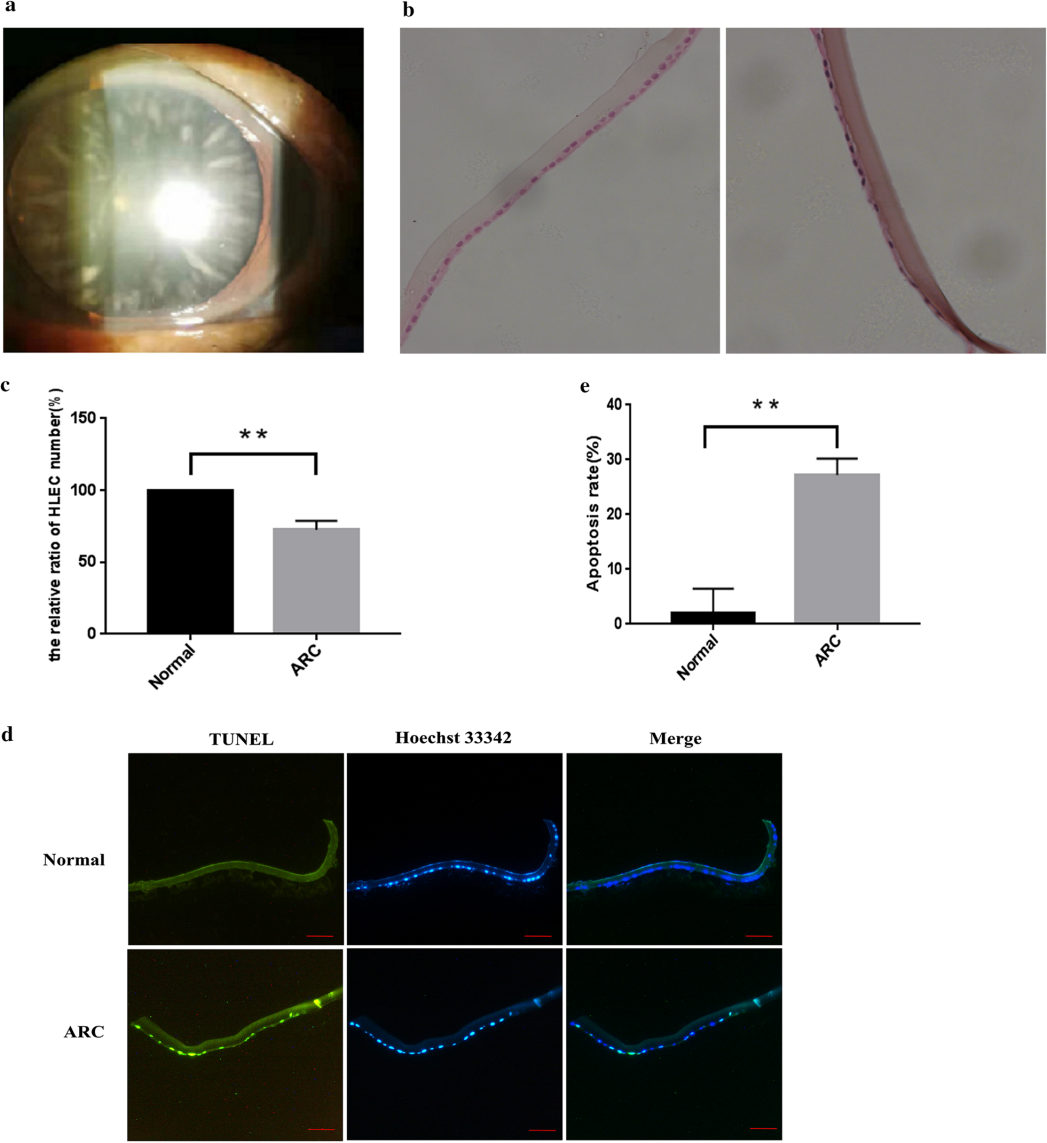
Lens epithelial cells apoptosis was associated with the age-related cataract base on human samples. **a** Representative slit-lamp image of the lens with cortical opacity of cataract. From the representative patient, the slit lamp biomicroscopy examination showed the cortical cataract with age. Because the normal subjects with transparent lens were enrolled from corneal transplant donors and ocular trauma patients with lens detachment, a picture of normal control cannot be obtained. **b** Decreases in LECs density from the cataract patients compared to the control individuals using HE staining of whole lenses. The patient lens epithelial cells showed cell nuclei shrinkage, chromatin condensation, and fragmented nuclei by nuclei stained with hematoxylin. Scale bar: 20 µm. **c** The cell density was determined by counting nuclei stained with the hematoxylin in 5 randomly selected fields from three experiments and are shown as means ± SD. **p < 0.01 versus the LECs from control individuals. **d** Apoptotic LECs increased in ARC patients. The red arrows point to the apoptotic cells with both positive TUNEL and Hoechst 33342 staining. The yellow arrows point to the non-apoptotic cells with TUNEL-negative staining. Scale bar: 100 µm. **e** The apoptotic cell numbers were determined by TUNEL assay. Counting both positive TUNEL and Hoechst 33342 cells in 5 randomly selected fields from three experiments and are shown as means ± SD. **p < 0.01 versus the LECs from control individuals

The clinical samples were obtained for studying morphology and mechanisms of ARC. The ARC representative picture is shown in Fig. 1a. From the representative patient, the slit lamp biomicroscopy examination showed the cortical cataract with age. HE staining assay showed that the nucleus of normal lens epithelial cells was round and arranged relatively uniformly, while the cell nucleus of ARC patients was constricted and arranged unevenly, and the number of cells was reduced (Fig. 1b). Moreover, the numbers of lens epithelial cells from the patient were about 28% less than the control individuals (Fig. 1c). The TUNEL assay showed that the apoptotic epithelial cells were 25% in the lens from cataract patients more than that in the control individuals (Fig. 1d, e).

### Lens epithelial cells apoptosis was associated with the age-related cataract base on mice samples

**Fig. 2:**
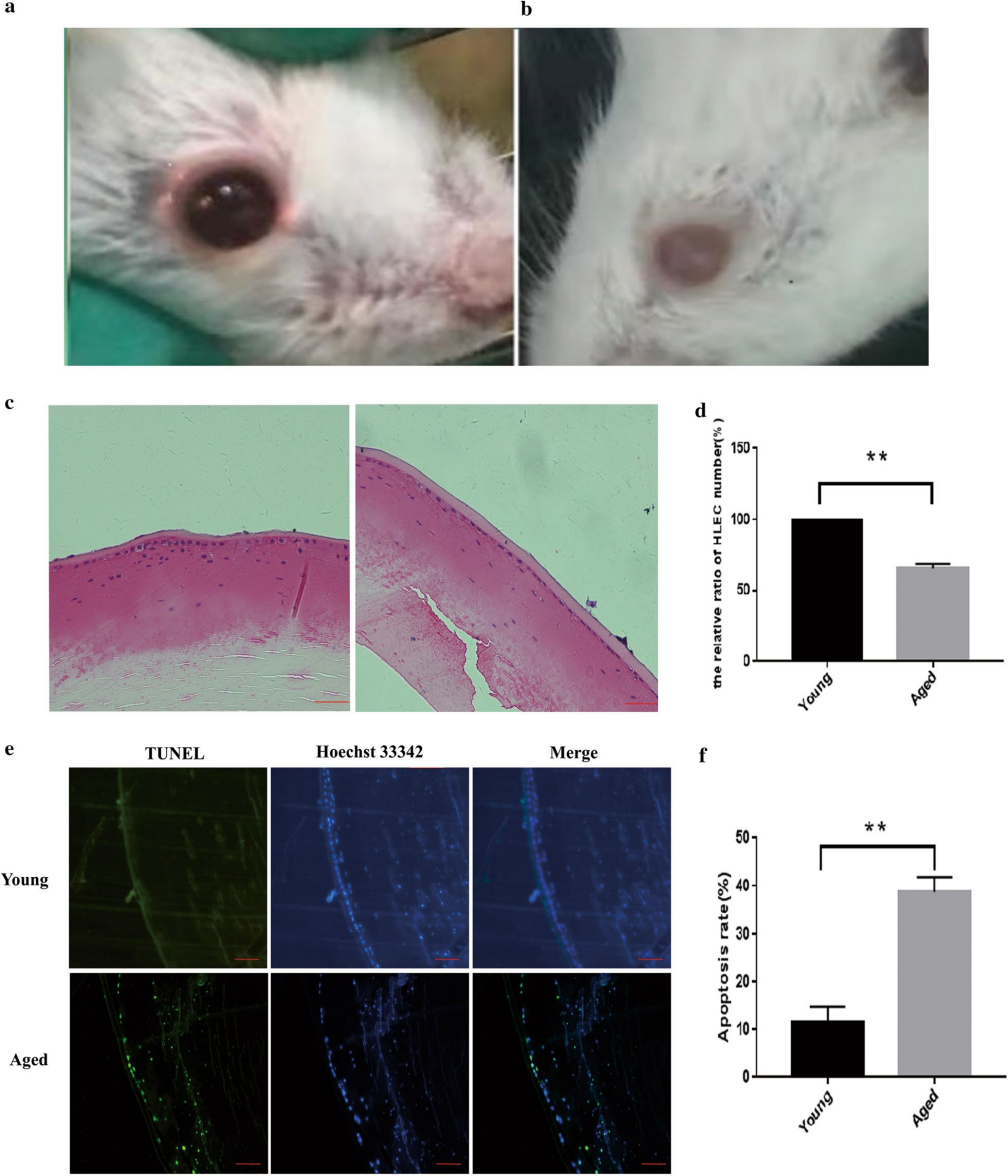
Lens epithelial cells apoptosis was associated with the age-related cataract base on mice samples. **a**, **b** Displayed the young and aged eye photos respectively. The eyes of the young (3 months) mice remained clear, while, the natural aging mice displayed obvious cataracts over 24 months, and the eyes photos were taken. **c** A comparison of the lens epithelial cells between the old and young mice. The LECs stained with eosin, nuclei stained with hematoxylin was shown in blue. Scale bar: 20 µm. **d** Decreases in mouse LECs density from the old mice compared to the young mice using HE staining of whole lenses. The cell density was determined by counting nuclei stained with the hematoxylin in 5 randomly selected fields from three experiments and are shown as means ± SD. **p < 0.01 versus the young LECs. **e** Apoptotic LECs increased in old mice by positive TUNEL and Hoechst 33342 staining. Scale bar: 100 µm. **f** Counting both positive TUNEL and Hoechst 33342 cells in 5 randomly selected fields from three experiments and are shown as means ± SD. **p < 0.01 versus the young LECs

The mice were used for further study. As shown in Fig. 2a, the light in the pupil area of the eye of young mice was red, meaning the lens was clear. In naturally age mice, the pupil area of the eye was white, indicating the development of cataracts (Fig. 2b). HE staining showed that the LECs of young mice were more uniformly arranged, while that of the aged mice was unevenly arranged and the number of cells was significantly reduced. Besides, the LECs of aged mice exhibited apoptotic characteristics such as nuclear contraction, nuclear fragmentation, and chromatin concentration (Fig. 2c, d). TUNEL assay showed that the apoptotic numbers of LECs from aged mice increased than the young mice (Fig. 2e, f), indicating the LECs from aged mice tend to go to apoptosis. Base on the human and mice experiments, we found that LECs apoptosis was associated with the ARC.

### Up-regulation of ROCK1 in the lens epithelial cells from age-related cataract patients and the old mice respectively

**Fig. 3:**
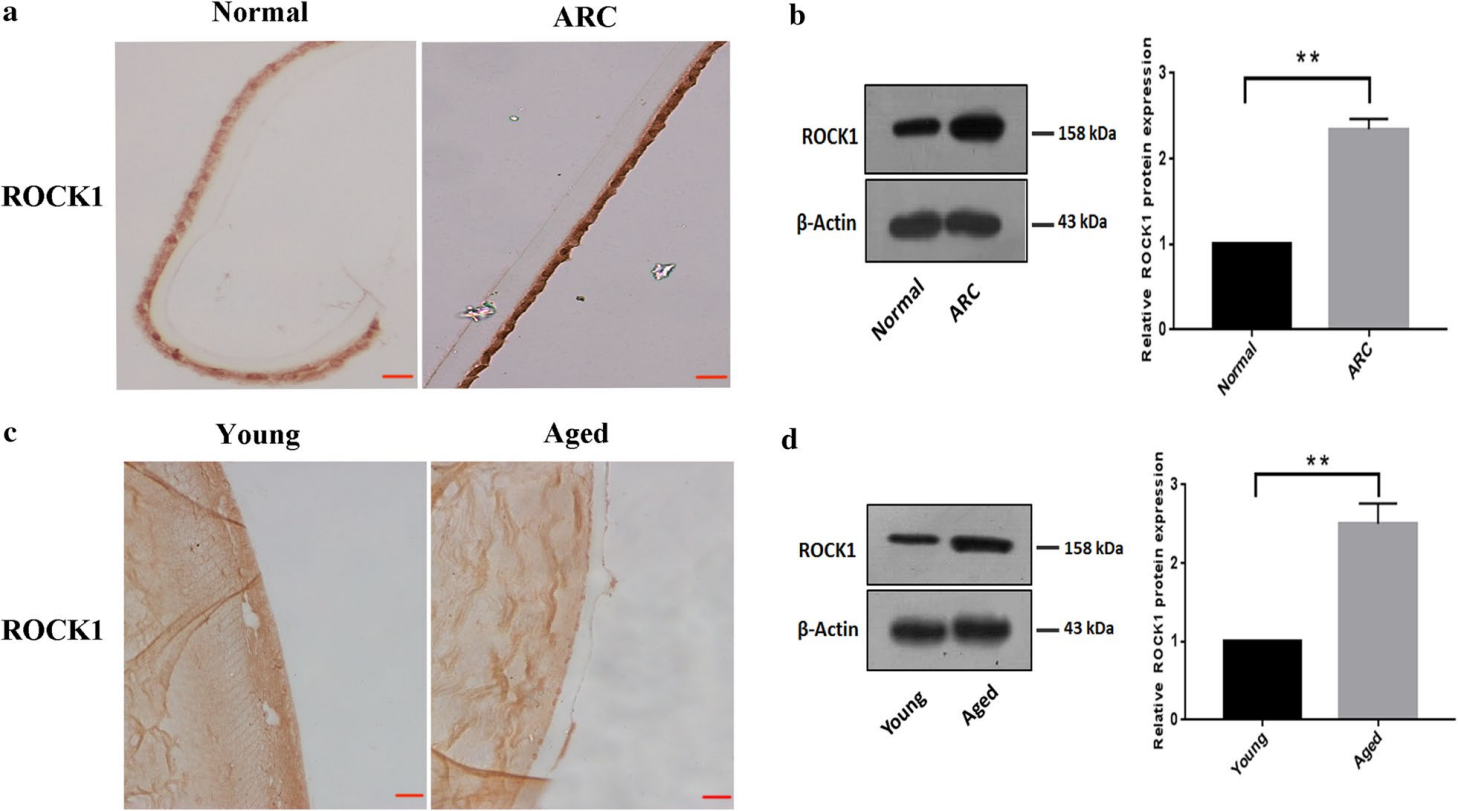
Up-regulation of ROCK1 in the lens epithelial cells from age-related cataract patients and the old mice respectively. **a** Up-regulation of ROCK1 in LECs from cataract patients compared to the control group. Lens sections were immunostained to assess the ROCK1 expression in the LECs from the patient and control group. Scale bar: 20 µm. **b** Western blotting estimated that the protein level of ROCK1 between the patient and the control group. The 100–250 kDa blot was retained by horizontal shear and incubated with the ROCK1 antibody. The 35–50 kDa blot was retained by horizontal shear and incubated with the β-actin antibody. **c** Lens sections were immunostained to assess the ROCK1 expression in the LECs from young and old mice, Scale bar: 20 µm. **d** Western blotting estimated that the protein level of ROCK1 between young and old mice, β-actin was used as an internal reference control. The 100–250 kDa blot was retained by horizontal shear and incubated with the ROCK1 antibody. The 35–50 kDa blot was retained by horizontal shear and incubated with the β-actin antibody

Immunohistochemistry showed that the protein level of ROCK1 was increased in LECs from cataract patients compared to the control group (Fig. 3a). Moreover, western blotting indicated that ROCK1 expression was increased in the LECs of cataract patients (Fig. 3b). Meanwhile, Immunohistochemistry showed that the protein level of ROCK1 was increased in LECs from old mice than the young mice (Fig. 3c). Western blotting also confirmed that increased protein levels of ROCK1 in old mice compared to the control group (Fig. 3d). Taken together, the up-regulation of ROCK1 may be relative to the ARC.

### Up-regulation of ROCK1 in apoptotic epithelial cells in response to H_2_O_2_

**Fig. 4:**
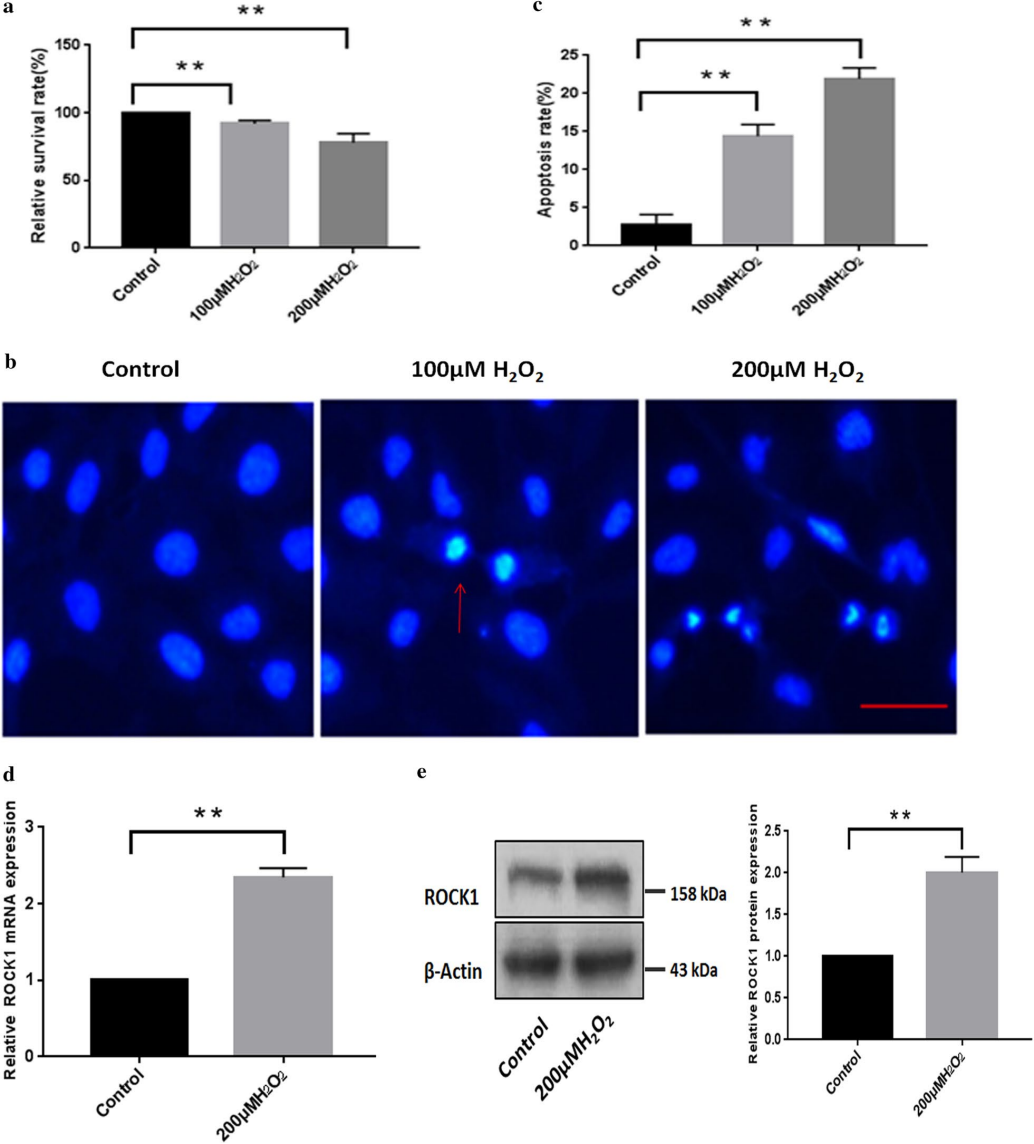
Up-regulation of ROCK1 in apoptotic epithelial cells in response to H_2_O_2_. **a** H_2_O_2_ inhibited the viability of LECs as measured by the MTT assays. B3 cells were incubated in the absence or presence of different doses of H_2_O_2_ (100 and 200 µM) for 24 h, and cell viability was assessed by measuring absorbance at 492 nm with a microplate reader. **p < 0.01 versus the untreated cells. **b** The apoptosis detection of LECs under different doses of H_2_O_2_ (100 and 200 µM) treatment for 24 h by Hoechst 33342 staining assay. The red arrow showed the nuclear morphology stained with blue by using Hoechst 33342. Scale bar: 50 µm. **c** Apoptosis rates were analyzed after treatment with different doses of H_2_O_2_ (100 and 200 µM) treatment. The apoptosis rates, which were calculated based on at least 100 cells from three experiments, **p < 0.01 versus the untreated cells. **d** The mRNA expression of ROCK1 in B3 cells under 200 µM H_2_O_2_ was assessed by quantitative RT-PCR. **p < 0.01 versus the untreated group. **e** The protein level of ROCK1 increased in lens epithelial cells under 200 µM H_2_O_2_ treatment. The 100–250 kDa blot was retained by horizontal shear and incubated with the ROCK1 antibody. The 35–50 kDa blot was retained by horizontal shear and incubated with the β-actin antibody

Then, we aimed to identify the biological role of the ROCK1 gene in LECs in vitro. First, we investigated the fate of HLE-B3 cells after treatment with different concentrations of H_2_O_2_. B3 cells were incubated in the absence or presence of different doses of H_2_O_2_ (100 and 200 µM) for cell survival assay. MTT assays showed that high H_2_O_2_ (200 µM) was able to decrease the survival percentage of B3 cells (Fig. 4a). We then found that the apoptotic cells were markedly increased in B3 cells in response to H_2_O_2_ treatment (Fig. 4b), the apoptosis rate was about sixfold that of the control (Fig. 4c). Then we assessed the expression of ROCK1 by comparing control cells and H_2_O_2_-treated cells using quantitative RT-PCR. As shown in Fig. 4d, the average expression of ROCK1 in H_2_O_2_ induced apoptotic cells was higher than the ROCK1 expression in untreated cells. The western blotting assay showed that the protein expression level of ROCK1 increased in B3 cells after H_2_O_2_-treatment (Fig. 4e). These results indicated that the up-regulation of ROCK1 was associated with LECs apoptosis in response to H_2_O_2_.

### ROCK1 plays a key role in mediating H_2_O_2_-induced lens epithelial cells apoptosis

**Fig. 5:**
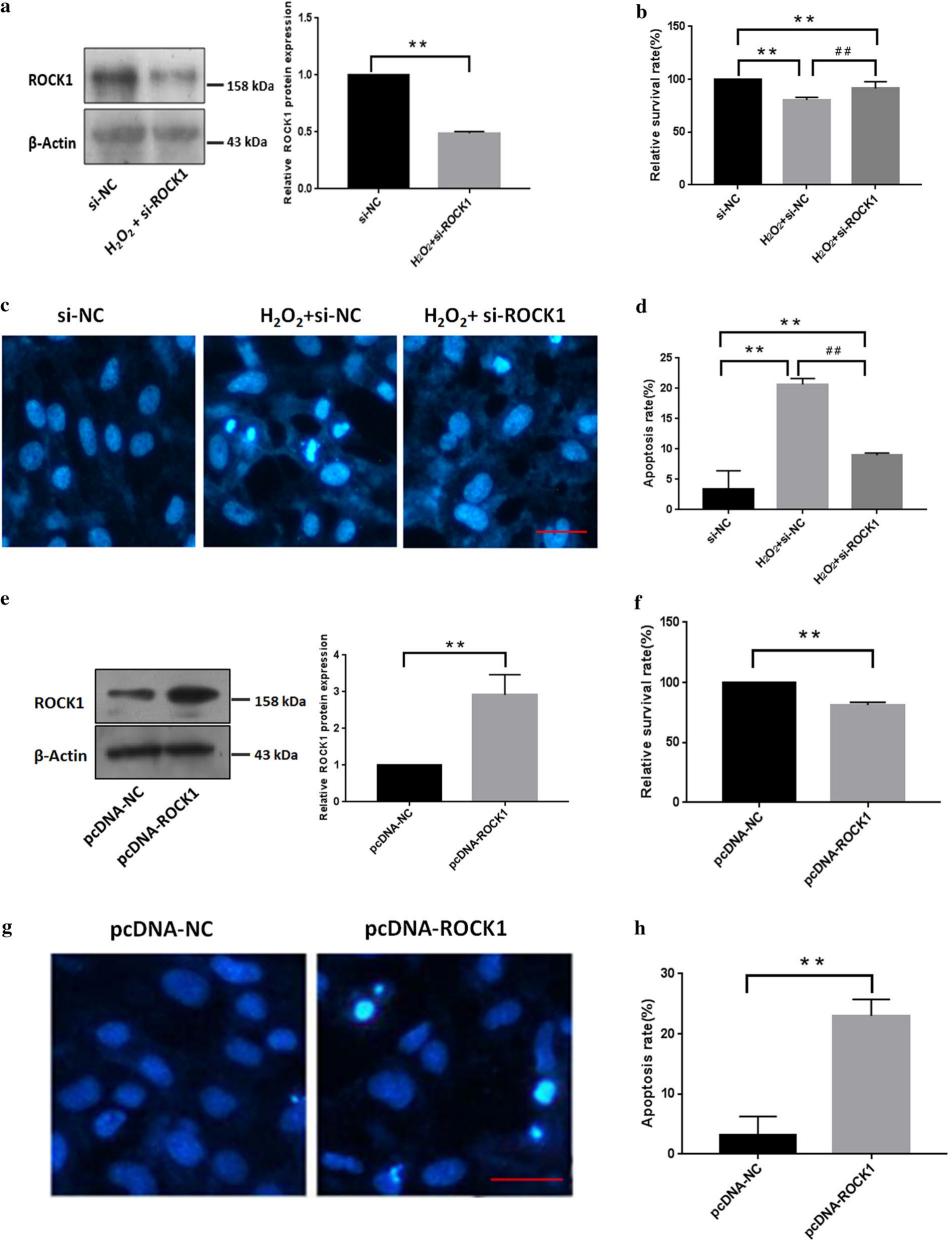
ROCK1 plays a key role in mediating H_2_O_2_-induced lens epithelial cells apoptosis. **a** Western blotting confirmation of the siRNA-mediated ROCK1 knockdown. Cells were lysed for western blotting after transfection with si-ROCK1 and si-NC, respectively. β-actin was used as an internal reference. Data are based on three independent experiments. The 100–250 kDa blot was retained by horizontal shear and incubated with the ROCK1 antibody. The 35–50 kDa blot was retained by horizontal shear and incubated with the β-actin antibody. **b.** Cells transfected with si-ROCK1 were treated with H_2_O_2_ for 24 h and cell survival was tested by the MTT assay (n = 5). **p < 0.01 versus untreated group. ^##^p < 0.01 versus H_2_O_2_-treated group. Data are based on three independent experiments. **c** ROCK1-knockdown suppressed H_2_O_2_-induced cell apoptosis by apoptotic assay. Cells transfected with si-ROCK1 were treated with H_2_O_2_ for 24 h, and cell apoptosis was visualized by Hoechst 33342 staining assay. **d** Cell apoptosis was analyzed after transfection with si-ROCK1 plasmids. **p < 0.01 versus untreated group. ^##^p < 0.01 versus H_2_O_2_-treated group. Scale bar: 50 µm. **e** Western blotting confirmation of the over-expression levels of ROCK1 after transfection with the pcDNA-ROCK1 plasmid. The control group was transfection with the pcDNA-NC plasmid. The 100–250 kDa blot was retained by horizontal shear and incubated with the ROCK1 antibody. The 35–50 kDa blot was retained by horizontal shear and incubated with the β-actin antibody. **f** ROCK1 over-expression decreased B3 cell survival was determined by MTT analysis. Cell survival was tested after transfection with pcDAN-ROCK1 plasmids by the MTT assay (n = 5). **p < 0.01 versus untreated group. **g** ROCK1 over-expression induced cell apoptosis in B3 cells by apoptotic assay. Scale bar: 50 µm. **h** Cell apoptosis was analyzed after transfection with ROCK1 overexpression plasmids. **p < 0.01 versus the untransfected group

To clarify the role of ROCK1 in H_2_O_2_-induced LECs apoptosis, we constructed a si-ROCK1 plasmid. Western blotting showed that the transfection of B3 cells with si-ROCK1 could significantly reduce ROCK1 protein levels (Fig. 5a). MTT assay showed that knockdown of endogenous ROCK1 increased cell viability compared to cells treated with H_2_O_2_ alone (Fig. 5b). Correspondingly, it also caused a decrease in the numbers of apoptotic cells (Fig. 5c, d). Then, we used the pcDNA-ROCK1 plasmid for further study. After transfecting the pcDNA-ROCK1 plasmid into B3 cells, ROCK1 expression was significantly increased by western blotting assay. Meanwhile, MTT assay showed that overexpression of ROCK1 inhibited LECs survival (Fig. 5f). Apoptotic assays showed that overexpression of ROCK1 led to the apoptosis of LECs (Fig. 5g, h).

### Up-regulation of p53 is associated with ROCK1-mediated lens epithelial cells apoptosis in response to H_2_O_2_ treatment

**Fig. 6:**
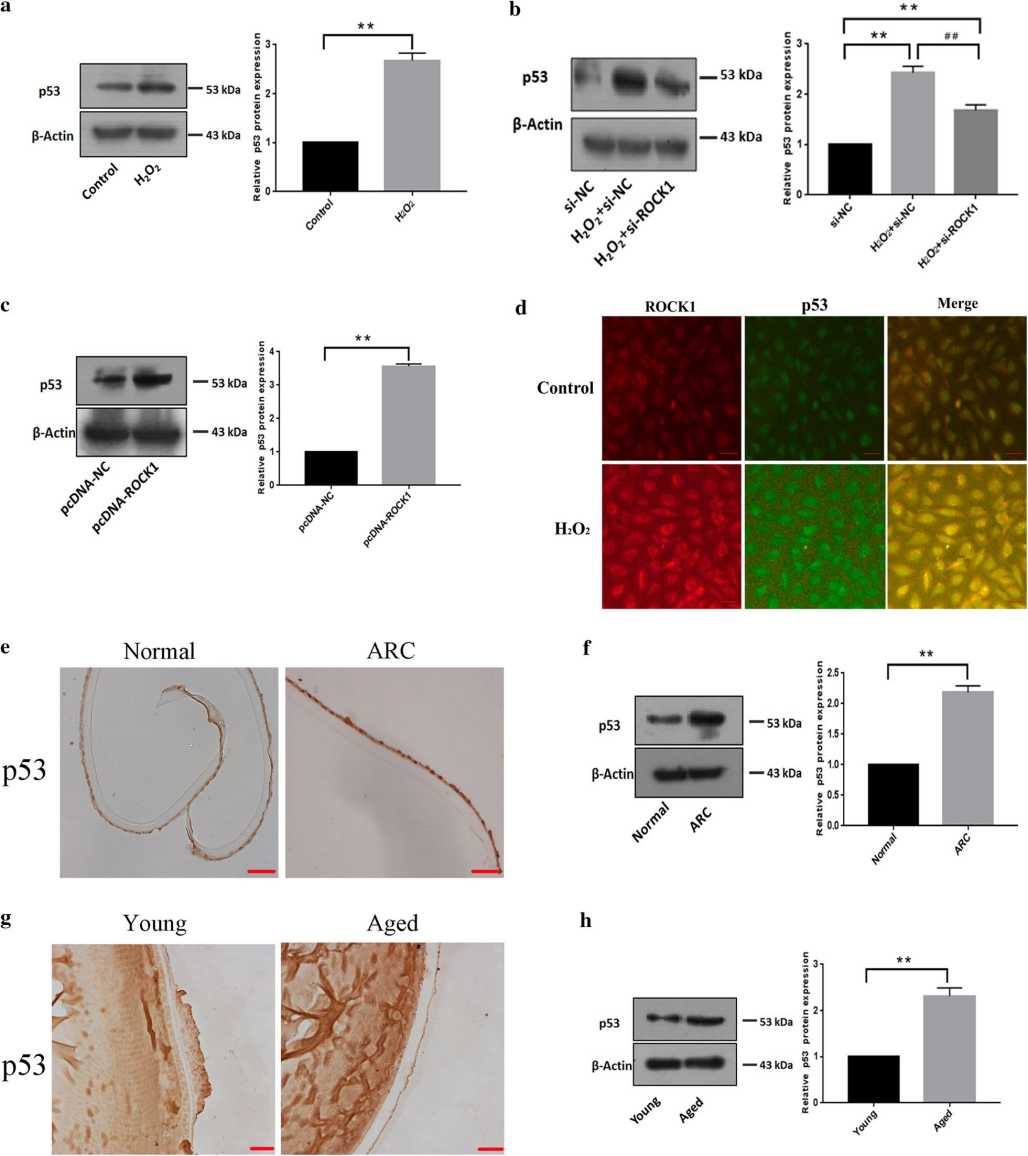
Up-regulation of p53 is associated with ROCK1-mediated lens epithelial apoptosis in response to H_2_O_2_ treatment. **a** The protein level of p53 increased in epithelial cells after H_2_O_2_ treatment for 24 h. The 40–70 kDa blot was retained by horizontal shear and incubated with a p53 antibody. The 35–50 kDa blot was retained by horizontal shear and incubated with the β-actin antibody. **b** The knock-down of ROCK1 suppressed the increase in protein levels of p53 in response to H_2_O_2_. LECs transfected with si-ROCK1 were treated with H_2_O_2_ for 24 h and then lysed for western blotting. Data are based on three independent experiments. The 40–70 kDa blot was retained by horizontal shear and incubated with a p53 antibody. The 35–50 kDa blot was retained by horizontal shear and incubated with the β-actin antibody. **c** The protein level of p53 increased in B3 cells after transfection with ROCK1 over-expression plasmid. The 40–70 kDa blot was retained by horizontal shear and incubated with a p53 antibody. The 35–50 kDa blot was retained by horizontal shear and incubated with the β-actin antibody. **d** Immunofluorescence showed that ROCK1 and p53 coexist in the cytoplasm, and the co-localization expression of ROCK1 and p53 increased under the induction of H_2_O_2_. Scale bar: 100 µm. **e** Immunohistochemistry showed that the protein level of p53 in lens epithelial cells from the cataract patient and control group (n = 5 per group). Scale bar: 20 µm. **f** Western blotting indicated that p53 expression was increased in lens epithelial of cataract patients compared to the control group. The 40–70 kDa blot was retained by horizontal shear and incubated with a p53 antibody. The 35–50 kDa blot was retained by horizontal shear and incubated with the β-actin antibody. **g** Lens sections were immunostained to assess the p53 expression in the LECs from young and old mice, Scale bar: 20 µm. **h** Western blotting p53 expression was increased in lens epithelial from young and old mice, (n = 5 per group). β-actin was used as an internal reference control. The 40–70 kDa blot was retained by horizontal shear and incubated with a p53 antibody. The 35–50 kDa blot was retained by horizontal shear and incubated with the β-actin antibody

Western blotting showed that the up-regulation of p53 protein levels in LECs after H_2_O_2_ treatment (Fig. 6a). Increases in p53 expression in response to H_2_O_2_ were remarkably attenuated when the ROCK1 expression was knocked down using the RNAi (Fig. 6b). Western blotting also showed that the protein level of p53 increased after transfection with the pcDNA-ROCK1 plasmid (Fig. 6c). Immunofluorescence showed ROCK1 and p53 coexist in the cytoplasm, and the co-localization expression of ROCK1 and p53 increased under the induction of H_2_O_2_ (Fig. 6d). Immunohistochemistry showed that the protein level of p53 was increased in LECs from cataract patients compared to the control group (Fig. 6e). Western blotting indicated that p53 expression was increased in LECs from the patient than the control group (Fig. 6f). Meanwhile, Immunohistochemistry showed that the protein level of p53 was increased in LECs from old mice than the young mice (Fig. 6g). Western blotting also confirmed that increased protein levels of p53 in old mice compared to the control group (Fig. 6h).

### p53 participated in ROCK1-mediated lens epithelial cells apoptosis in response to H_2_O_2_ treatment

**Fig. 7:**
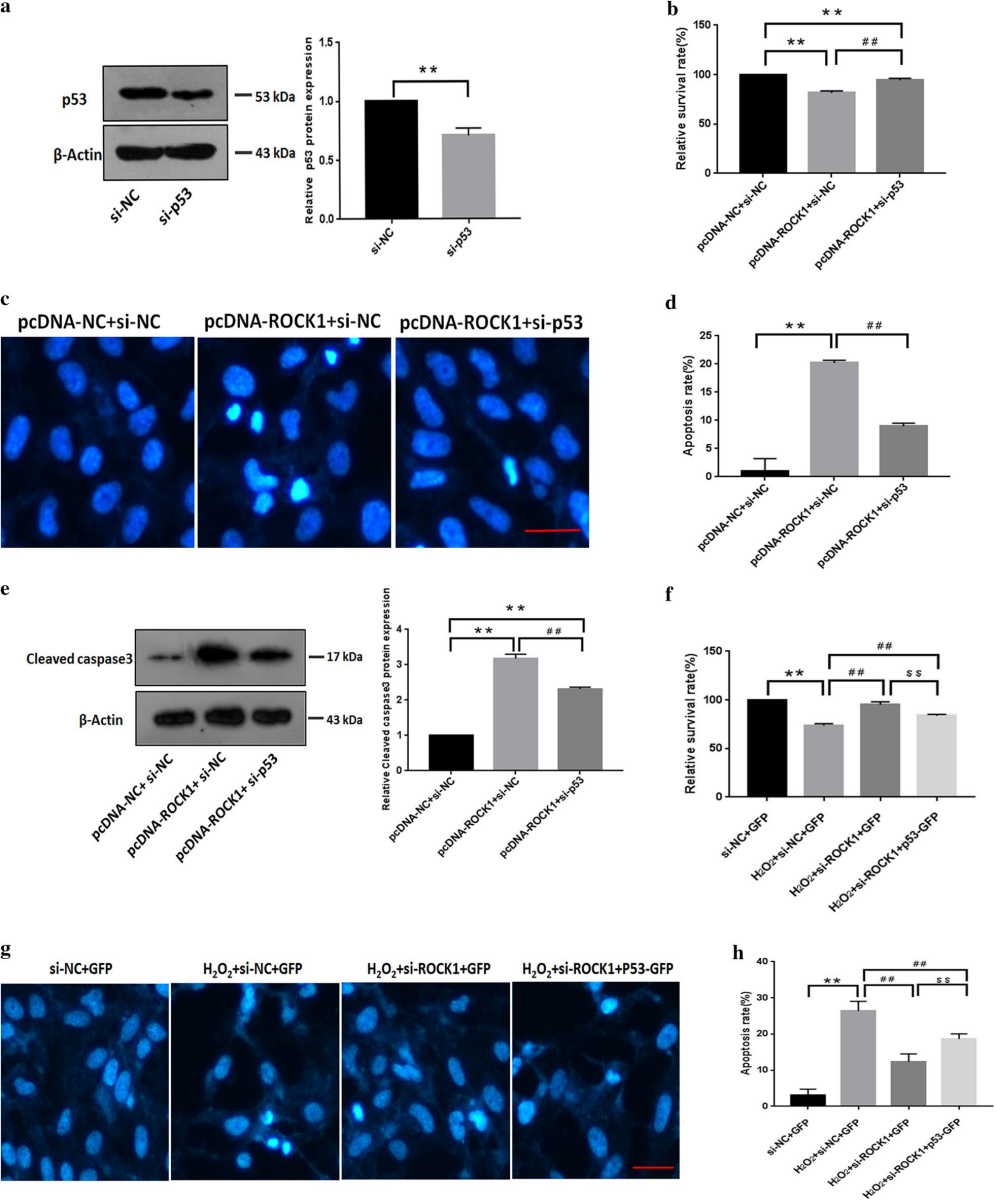
p53 participated in ROCK1-mediated lens epithelial cells apoptosis in response to H_2_O_2_ treatment. **a** Western blotting confirmation of the siRNA-mediated p53 knockdown. Cells were lysed for western blotting after transfection with si-p53 and si-NC, respectively. β-actin was used as an internal reference. Data are based on three independent experiments. The 40–70 kDa blot was retained by horizontal shear and incubated with a p53 antibody. The 35–50 kDa blot was retained by horizontal shear and incubated with the β-actin antibody. **b** The knockdown of p53 resisted the decreased cell survival index after ROCK1 over-expression. Cells transfected with the pcDNA-ROCK1 and si-p53 and were tested by the MTT assay. **p < 0.01 versus the pcDNA-NC and si-NC group. ^##^p < 0.01 versus pcDNA-ROCK1 and si-NC group, (n = 3). **c** The knockdown of p53 resisted the increase in apoptotic cells after ROCK1 over-expression. The apoptosis detection of LECs after transfection with pcDNA-ROCK1 and si-p53. Scale bar: 50 µm. **d** The apoptosis rates, which were calculated based on at least 100 cells from three experiments, **p < 0.01 versus the pcDNA-NC and si-NC group. ^##^p < 0.01 versus pcDNA-ROCK1 and si-NC group. **e** Western blotting indicated that knockdown of p53 suppressed the increase in cleaved caspase3 levels by ROCK1 overexpression. The 10–30 kDa blot was retained by horizontal shear and incubated with a cleaved caspase3 antibody. The 35–50 kDa blot was retained by horizontal shear and incubated with the β-actin antibody. **f** Cells transfected with si-ROCK1 and p53-GFP were treated with H_2_O_2_ for 24 h and cell survival was tested by the MTT assay (n = 5). **p < 0.01 versus si-NC and GFP group. ^##^p < 0.01 versus si-NC and GFP under H_2_O_2_-treated group. ^$$^p < 0.01 versus si-ROCK1 and GFP under H_2_O_2_-treated group. Data are based on three independent experiments. **g** Cells transfected with si-ROCK1 and p53-GFP were treated with H_2_O_2_ for 24 h and cell apoptosis was visualized by the Hoechst 33342 staining assay. **h** Cell apoptosis was analyzed after transfection with si-ROCK1 and p53-GFP plasmids. **p < 0.01 versus si-NC and GFP group. ^##^p < 0.01 versus si-NC and GFP under H_2_O_2_-treated group. ^$$^p < 0.01 versus si-ROCK1 and GFP under H_2_O_2_-treated group. Scale bar: 50 µm

We examined whether the p53 participates in ROCK1-mediated apoptosis, si-p53 plasmid was constructed, and western blotting confirmed that the protein expression of p53 decreased markedly after the transfection of si-p53 plasmid (Fig. 7a). MTT assay showed that p53 inhibition partially restored ROCK1-induced inhibition of epithelial cells^**’**^ survival (Fig. 7b). Suppression of endogenous p53 reduced ROCK1-induced LECs apoptosis (Fig. 7c, d). Western blotting showed that the expression of cleaved caspase3 increased when ROCK1 was overexpressed, and it was down-regulated when ROCK1 overexpression combined with p53 silence (Fig. 7e). MTT assay showed that the proliferation rate of cells increased after the silent expression of ROCK1, and the proliferation rate decreased after the over-expression of p53 (Fig. 7f). Apoptotic assays showed that in the environment of H_2_O_2_ induced apoptosis, the apoptotic cells will be significantly reduced after ROCK1 is silenced, and overexpression of p53 can partially inhibit the reduction of apoptosis rate caused by ROCK1 silencing (Fig. 7g, h).

### p53 (ser15) phosphorylation was increased in ROCK1-mediated lens epithelial cells apoptosis in response to H_2_O_2_ treatment

**Fig. 8:**
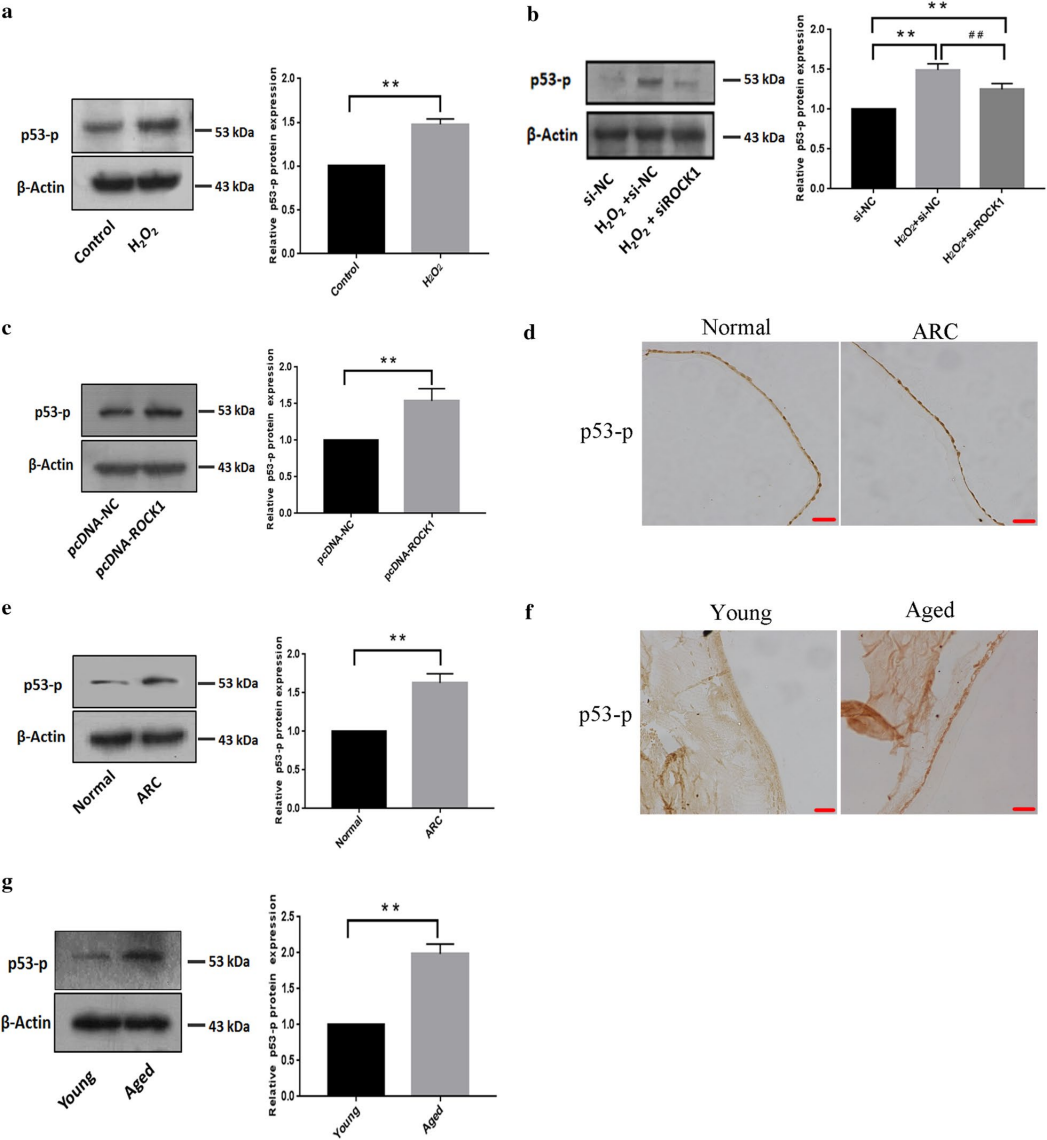
p53 (ser15) phosphorylation was increased in ROCK1-mediated lens epithelial cells apoptosis in response to H_2_O_2_ treatment. **a** The p53 (ser15) phosphorylation level increased in epithelial cells after H_2_O_2_ treatment for 24 h. The 40–70 kDa blot was retained by horizontal shear and incubated with a p53-p antibody. The 35–50 kDa blot was retained by horizontal shear and incubated with the β-actin antibody. **b** The knockdown of ROCK1 suppressed the increase in phosphorylation levels of p53 (ser15) in response to H_2_O_2_. LECs transfected with ROCK1 siRNA were treated with H_2_O_2_ for 24 h and then lysed for western blotting. Data are based on three independent experiments. The 40–70 kDa blot was retained by horizontal shear and incubated with a p53-p antibody. The 35–50 kDa blot was retained by horizontal shear and incubated with the β-actin antibody. **c** The p53 (ser15) phosphorylation level increased in B3 cells after transfection with ROCK1 over-expression plasmid. The 40–70 kDa blot was retained by horizontal shear and incubated with a p53-p antibody. The 35–50 kDa blot was retained by horizontal shear and incubated with the β-actin antibody. **d** Immunohistochemistry showed that the p53 (ser15) phosphorylation level in lens epithelial cells from cataract patient and control group (n = 5 per group). Scale bar: 20 µm. **e** Western blotting indicated that the p53 (ser15) phosphorylation level increased in lens epithelial of cataract patient compare to the control group. The 40–70 kDa blot was retained by horizontal shear and incubated with a p53-p antibody. The 35–50 kDa blot was retained by horizontal shear and incubated with the β-actin antibody. **f** Lens sections were immunostained to assess the p53 (ser15) phosphorylation level in the LECs from young and old mice, Scale bar: 20 µm. **g** Western blotting showed that the p53 (ser15) phosphorylation level was increased in lens epithelial from young and old mice, (n = 5 per group). β-actin was used as an internal reference control. The 40–70 kDa blot was retained by horizontal shear and incubated with a p53-p antibody. The 35–50 kDa blot was retained by horizontal shear and incubated with the β-actin antibody

Our results showed that increased phosphorylation of p53 in at ser15 in B3 cells after H_2_O_2_-treatment (Fig. 8a). Increases in p53 (ser15) phosphorylation in response to H_2_O_2_ were remarkably attenuated when the ROCK1 expression was knocked down using the RNAi (Fig. 8b). Moreover, western blotting showed that phosphorylation of p53 at ser15 increased after transfection with pcDNA-ROCK1 (Fig. 8c). Immunohistochemistry showed that the p53 (ser15) phosphorylation was increased in LECs from cataract patients compared to the control group (Fig. 8d). Western blotting indicated that p53 (ser15) phosphorylation was increased in LECs of cataract patients compared to the control group (Fig. 8e). Meanwhile, Immunohistochemistry showed that the p53 (ser15) phosphorylation was increased in lens epithelial cells from old mice than the young mice (Fig. 8f). Western blotting also confirmed that increased protein levels of p53 (ser15) phosphorylation in old mice compared to the young mice (Fig. 8g).

## Discussion

Age-related cataracts result in severe visual impairment, lower quality of life, which has a significant impact on health resources and social economy. A poor understanding of the pathogenesis of cataracts has been a hurdle in such a pursuit. Our results showed apoptosis in LECs plays a vital role in the pathogenesis of ARC through the clinic, mice experiment, and cytologic study. The research identified that ROCK1 was up-regulated in the LECs from ARC patients and the old mice compared with control groups, respectively. Meanwhile, this paper also found that ROCK1 was up-regulated in HLE-B3 cells in response to H_2_O_2_. Besides, we found ROCK1 played a key role in mediating H_2_O_2_-induced apoptosis of LECs through ROCK1 over-expression and knockdown experiments, respectively. Further investigation showed that p53 protein levels increased during ROCK1-mediated apoptosis in response to H_2_O_2_. Moreover, our results showed that ROCK1 mediated the p53 phosphorylation ser15 to up-regulate its protein level. Data from this study established the novel association of ROCK1/p53 signaling with LECs apoptosis and ARC.

Apoptosis, a genetically programmed cell death, is the final result of biochemical pathways under the influence of stimuli such as oxidative stress, high temperature, and ionizing radiation et al. (Li et al. 1995). Apoptotic cells, also called apoptotic bodies, are morphologically characterized by cell shrinkage, nuclear chromatin condensation, and dense cytoplasmic organelles (Charakidas et al. 2005). In the present study, our results showed that the numbers of LECs from patients were less than the control individuals. And the TUNEL assay showed that the apoptotic epithelial cells were significantly increased in the lens from cataract patients compared to control through the human and mice experiments. The results suggested that LECs apoptosis may play a role in the pathogenesis of ARC, which is consistent with previous studies from Charakidas et al. (2005) and Osnes-Ringen et al. (2016). In vitro, by incubating in the absence or presence of different doses of H_2_O_2_ (100 and 200 µM), the research established a model of lens epithelial cell apoptosis induced by oxidative stress. The results showed that the apoptotic cells were markedly increased in B3 cells in response to H_2_O_2_ (200 µM) treatment, the apoptosis rate was about sixfold that of the control. These results above showed that oxidative stress could trigger LECs apoptosis that then might initiate cataract development. Thus, an understanding of the molecular mechanism of H_2_O_2_ induced LECS apoptosis may be required to open the door for new therapeutic strategies.

RhoA/ROCK signaling has been reported involved in different eye diseases which is an important molecular “switch”. Nakamura et al. (2001) have shown that ROCK participated in the migration of rabbit corneal epithelial cells by regulating the actin cytoskeleton. A study by Rao et al. (2001) showed that RhoA inhibitors downregulate the functions of the actin cytoskeleton and myosin light-chain phosphatase, causing relaxation of trabecular meshwork and Schlemm’s canal cells, reducing cell adhesion with underlying cells, increasing outflow pathways of aqueous humor, and reducing the outflow resistance of aqueous humor. Thus, RhoA can be used as a new target for the development of glaucoma medications. RhoA/ROCK signaling is also involved in the dysfunction of retinal pigment epithelial cells (Ruiz et al. 2011). However, the expression and role of ROCK1 in LECs in exposure to H_2_O_2_ had not been revealed, furthermore, the relation between ROCK1 and ARC has not been reported. Our results showed that the protein levels of ROCK1 increased in the lens epithelial cells from age-related cataract patients, old mice, and H_2_O_2_-induced LECs respectively. Besides, the inhibition of LECs survival induced by H_2_O_2_ was partly resisted by ROCK1 knockdown, and the ROCK1 overexpression decreased the cell survival of epithelial cells and led to the apoptosis of LECs. Based on the experimental data generated from this study, it established the association ROCK1 up-regulation with LECs apoptosis and ARC. In our study, we found that ROCK1 plays an important role in the occurrence of ARC and is expected to become a target for treatment.

Selective inhibition of a signaling pathway to treat diseases has been widely studied as a potential approach. It has been reported that the inhibition of ROCK has a therapeutic effect on the field of ophthalmology. As a feasible treatment for glaucoma, ROCK inhibitor eye drops (netarsudil) have been performed in clinical trials (Sturdivant et al. 2016). Also, ROCK inhibitor (ripasudil) can significantly prevent retinal edema, reduce the size of the non-perfused area, improve retinal blood flow, which is a potential therapeutic agent for retinal vein occlusion (Hida et al. 2018). For the lens of eyes, researchers have found that the application of a ROCK inhibitor (Y-27632) can prevent posterior capsule opacification, and anterior subcapsular cataract induced by UV-B irradiation (Imaizumi et al. 2019; Lin et al. 2019). However, whether ROCK inhibitors can be used to treat age-related cataracts has not been studied, which is the direction of our upcoming research.

The p53 protein is a regulative factor in regulating cell cycle, cell differentiation, promoting apoptosis, and activating cell death (Wawryk et al. 2014). The protein level of p53 is controlled by post-translational modifications, such as phosphorylation. The most frequently described phosphorylation is on ser15 of p53 and occurs in response to different stress signals. Phosphorylation of p53 at the N-terminal residue ser15 is crucial for weakening interactions between p53 and Mdm2, a known negative regulator of p53, thereby stabilizing p53 (Bode and Dong 2004; Seong and Ha 2012). Thus, this paper examined the p53 (ser15) phosphorylation in this study. Our results showed that up-regulation of p53 and p53 (ser15) phosphorylation protein levels in HLE-B3 cells after H_2_O_2_ treatment, age-related cataracts patients, and old mice models compared with control groups. And the cytologic study showed the increases in p53 and p53 (ser15) phosphorylation in response to H_2_O_2_ were remarkably attenuated when the ROCK1 expression was knocked out using the RNAi. Meanwhile, the research showed p53 and p53 (ser15) phosphorylation in LECs also increased after transfection with ROCK1 expression plasmid. These results investigated the mechanism of ROCK1-mediated p53 and p53 (ser15) phosphorylation in LECs apoptosis and cataract. As the executor of apoptosis, caspase3 is also regulated by p53 (Xing et al. 2016; Wu et al. 2019; Sun et al. 2017). We found that the expression of cleaved caspase3 will increase when ROCK1 is overexpressed, and will be inhibited when p53 is silenced, which proves the key role of ROCK1/p53 signaling pathway in LECS apoptosis. Overall, this paper has experimentally demonstrated that phosphorylation at ser 15 was one of the important ways to regulate p53. These results demonstrate a new regulatory increase in the protein level of p53 in the occurrence of cataracts and ROCK1-mediated phosphorylation of p53 at ser15 which may lead to an increase in the protein level of p53 in the ARC.

## Conclusions

In the present study, our results showed that the numbers of LECs from patients were less than the control individuals and abnormal cell apoptosis may play a role in the pathogenesis of ARC through the clinic and mice experiment. Meanwhile, it revealed that the up-regulation of the ROCK1 gene was associated with H_2_O_2_-induced LECs apoptosis and ARC. Further investigation showed that p53 protein levels increased during ROCK1-mediated apoptosis in response to H_2_O_2_. Moreover, our results showed that ROCK1 mediated the p53 phosphorylation ser15 to up-regulate its protein level. Data from this study established the novel association of ROCK1/p53 signaling with LECs apoptosis and ARC. Future studies should be undertaken to develop drugs based on ROCK1 inhibitors, which can block this signal pathway, prevent, delay, and even cure the development of ARC.

## Data Availability

All data generated or analyzed during this study are included in this published article.

## Abbreviations

ARC: Age-related cataract
LECs: Lens epithelial cells
ROS: Reactive oxygen species
ROCK1: Rho-associated coiled-coil-containing protein kinase 1
HE: Hematoxylin and eosin
IHC: Immunohistochemical
PVDF: Polyvinylidene fluoride
HLE-B3: Human lens epithelial B3
FBS: Fetal bovine serum
RT-PCR: Reverse transcription-polymerase chain reaction
